# A New and Rare Presentation of Unilateral Recurrent Laryngeal Nerve Palsy in a COVID-19 Patient With No Recent History of Endotracheal Intubation

**DOI:** 10.7759/cureus.17700

**Published:** 2021-09-03

**Authors:** Swann Tin, Francine Foo, May Breitling, Jessie Saverimuttu, Christopher Lisi

**Affiliations:** 1 Internal Medicine, Richmond University Medical Center, Staten Island, USA; 2 Infectious Disease, Richmond University Medical Center, Staten Island, USA; 3 Head and Neck Surgery, Richmond University Medical Center, Staten Island, USA

**Keywords:** covid 19, recurrent laryngeal nerve palsy, intratracheal intubation, vocal cord dysfunction, systemic inflammatory response syndrome

## Abstract

The Coronavirus disease 2019 (COVID-19) infection has classical symptoms of high fevers, diarrhea, cough, and dyspnea; however, there are cases recording more unconventional features. In this case report, we will discuss recurrent laryngeal nerve palsy as a new and unusual presentation of COVID-19. The patient was a 58-year-old African American male with a history of hypertension, type-2 diabetes mellitus, and obstructive sleep apnea presenting with dyspnea, fatigue, and nausea. The patient was initially admitted to the medical intensive care unit (MICU) for acute hypoxic respiratory failure and completed intravenous Remdesivir for COVID-19. He never got intubated during the ICU stay and his condition improved on the 34th day of admission. However, two weeks later the patient suddenly developed hoarseness of voice. A bedside laryngoscopy revealed a left-sided vocal cord paralysis but patent airway. The computed tomography (CT) scan of the neck did not show any abnormalities, including any impinging masses or structures. The patient did not have any recent intubations to suggest the paralysis was due to traumatic injury, thus favoring that his neurologic injury was likely a post-viral symptom. One possible pathophysiology would be the invasion of nerve fibers (peripheral or cranial nerves) by the virus using the same mechanism as seen in alveolar cells and finally destroying them. Another hypothesis would be the inflammatory response of the host immune system affecting the peripheral and cranial nerves. Therefore, the potential association between neuro-invasiveness of severe acute respiratory syndrome coronavirus 2 (SARS-CoV-2) and the recurrent laryngeal nerve palsy resulting in the vocal cord paralysis should be considered and more studies need to be conducted for better understanding.

## Introduction

The Coronavirus disease 2019 (COVID-19) virus, first detected in Wuhan, China, was declared a pandemic on March 11, 2020 [1]. This highly infectious disease has affected millions of people worldwide and classic symptoms of high fevers, diarrhea, myalgia, cough and dyspnea have been well documented; however, there are cases recording more unconventional features [2]. Neurological manifestations of COVID-19 occur in roughly 36.4% of infected patients and mostly complain of myalgia and peripheral neuropathy [3]. In this case report, we will discuss recurrent laryngeal nerve palsy as a new and unusual presentation of COVID-19 and so far there are very few reported cases in the literature.

## Case presentation

A 58-year-old African American male with a history of hypertension, diabetes mellitus type 2, and obstructive sleep apnea presented to the Emergency Department with complaints of fatigue, nausea, and myalgias which began three weeks prior, and also dyspnea which began two days prior to presentation. The patient had his symptoms evaluated at an urgent care center 18 days prior and had a nasopharyngeal swab taken, the polymerase chain reaction (PCR) test was positive for COVID-19 infection. The patient is a never smoker and denied any history of respiratory diseases or autoimmune diseases.

On the day of admission, pulse rate was 120 beats per minute, blood pressure 142/96 mmHg, temperature 98.3 degrees Fahrenheit, and oxygen saturation 93% using 15L of supplemental oxygen via nonrebreather mask. He had a normal head inspection, with normal speech, no facial asymmetry, normal cardiovascular exam, and crackles in bilateral lungs were present on auscultation. Laboratory results were notable for a white blood cell count of 11.4 x 10^3/µL, creatinine 1.6 mg/dL, blood urea nitrogen 45 mg/dL, aspartate aminotransferase 50 U/L, alanine aminotransferase 102 U/L, hemoglobin a1c 7.8%, troponin 1.460 ng/mL, lactic acid, electrolytes, and arterial blood gas were within normal limits. COVID-19 PCR nasopharyngeal swab was also positive upon admission. A portable chest x-ray showed bilateral patchy infiltrates.

The patient was admitted to the medical intensive care unit and remained on strict airborne precautions. He required high concentrations of supplemental oxygen during the hospital course, which was delivered via high-flow nasal cannula and noninvasive positive pressure ventilation. The patient was treated with a one-time dose of intravenous remdesivir 200 mg, followed by 100 mg intravenously daily for four additional days, dexamethasone 6 mg for 10 days, doxycycline 100 mg tablets every 12 hours daily, and vitamin C 500 mg tablets daily, zinc 220 mg tablets daily, vitamin D 1,000 mg tablets daily. According to the Infectious Disease specialist’s recommendation on the eighth day of admission, the patient’s regimen was expanded to include baricitinib 4 mg one tablet daily for five days, and also includes broad-spectrum bacterial coverage with Aztreonam 1 g intravenously every eight hours and vancomycin 1 g intravenously every 12 hours (Table 1).

**Table 1:** Arterial blood gas (ABG) analysis showing pH, pCO2, pO2, HCO3, and blood gas modality throughout the patient's admission AVAPS - Average volume assured pressure support; BiPAP - Bilevel positive airway pressure

	On admission	1^st^ Day	8^th^ Day	12^th^ Day	13^th^ Day	24^th^ Day	36^th^ Day
Specimen type	Arterial	Arterial	Arterial	Arterial	Arterial	Arterial	Arterial
ABG pH	7.44	7.45	7.45	7.41	7.40	7.41	7.43
ABG pCO_2_	35	33	36	37	37	46	48
ABG pO_2_	81	97	48	73	100	76	135
ABG HCO_3_	23.8	22.9	25	23.5	22.9	29.2	31.9
ABG O_2_ Saturation	96.3	97.9	85.5	94.6	97.7	95.2	99.1
Blood Gas Modality	BiPAP	BiPAP	BiPAP	AVAPS	AVAPS	BiPAP	BiPAP
FiO_2_	90	100	100	100	70	100	100
Inspiratory BiPAP	14	14	16			14	14
Expiratory BiPAP	6	8	6			8	6

The patient's respiratory status began to improve and on the 34th day of admission, he was downgraded from the intensive care unit to the general medical floor. However, on the 43rd day of admission, the patient began to complain of hoarseness of voice. His symptom was not associated with dysphagia, stridor, or respiratory distress. On that day, the patient had a repeat nasopharyngeal swab done and PCR testing for COVID-19 was negative. The patient was evaluated by an ENT (ear-nose-throat) surgeon on day 48th of admission with a bedside laryngoscopy. The laryngoscopy revealed a left-sided vocal cord paralysis but patent airway. CT scan of the neck was performed which did not show any abnormalities, including any impinging masses or structures. The patient did not have any recent intubations to suggest the paralysis was due to traumatic injury, thus favoring that his neurologic injury was likely a post-viral symptom.

On day 52, the patient was stable to be discharged home. At the time, he was requiring 6L of supplemental oxygen which was arranged to continue upon discharge. Appointments for ENT follow-up were arranged. Upon the ENT appointment 46 days after discharge, he still presented with a hoarse voice. Therefore, he was offered vocal fold injection with Prolaryn Gel which he has chosen to pursue and will have the procedure done at a future date.

## Discussion

COVID-19 virus primarily infects the respiratory system by droplets or aerosol transmission. The mechanism by which the virus gains access into host cells is by attaching with its spike-like projections to angiotensin-converting enzyme-2 (ACE-2) on host cells and ACE-2 is found abundantly in alveolar cells of the lungs [4]. Being a positive-sense single-stranded RNA virus, it has the ability to complete a replication process inside the host cells. It is also thought to have virulence factors directed against host immune response. Most recent studies unravel that multiple structural and non-structural specifics of SARS-CoV-2, such as unique FURIN cleavage site, papain-like protease (SCoV2-PLpro), ORF3b and nonstructural proteins, and dynamic conformational changes in the structure of spike protein during host cell fusion, which give it an edge in infectivity and virulence over previous coronaviruses causing pandemics. COVID-19 affects different people in different ways. The most common symptoms are fever, dry cough, shortness of breath, chills, sore throat, headache, or chest pain. Less commonly it can cause gastrointestinal symptoms, loss of taste or smell, skin changes, confusion, and conjunctivitis. It also has been found to have some neurological complications such as cranial nerve palsy in some COVID-19 patients [5,6].

Vocal cord paralysis is a rare but severe complication after orotracheal intubation [7]. Vocal cord paralysis was seen in COVID-19 patients with endotracheal intubation most commonly due to traumatic as well as prolonged intubation periods resulting in compression of the recurrent laryngeal nerve between orotracheal tube cuff and the thyroid cartilage [8]. The most striking feature of our case report that makes it unique from all previous COVID-19 studies is that the patient suddenly and spontaneously developed vocal cord paralysis in his recovery period, with no previous or recent history of injury to his throat or intubation.

In COVID-19 patients who were never intubated, one possible pathophysiology would be the invasion of nerve fibers (peripheral or cranial nerves) by the virus using the same mechanism as seen in alveolar cells and finally destroying them [9,10] Another hypothesis would be the inflammatory response of the host immune system affecting the peripheral and cranial nerves [11,12]. Therefore, the potential association between the neuro-invasiveness of SARS-CoV-2 and the recurrent laryngeal nerve resulting in the vocal cord paralysis should be considered and more studies need to be conducted for better understanding.

## Conclusions

Based on no previous history of intubation, severe inflammatory response to COVID-19 throughout the hospital course and the late complication of unilateral laryngeal nerve palsy, especially after the full recovery of hypoxic respiratory failure strongly indicate the potential association of neuro-invasiveness of SARS-CoV-2. To date, speech therapy supports the recovery of voice change similar to the treatment of other vocal cord paralysis cases. Nevertheless, further studies should be performed for the association of possible SARS-CoV-2 neuro-invasiveness and recurrent laryngeal nerve palsy.
